# The Hematopoietic Function of Medicinal Wine Maoji Jiu Revealed in Blood Deficiency Model Rats

**DOI:** 10.1155/2022/1025504

**Published:** 2022-07-19

**Authors:** Yongli Xu, Fanqiang Zeng, Jianping Jiang, Juan Huo, Chengjian Zhao, Zhigang Yan, Li Li

**Affiliations:** ^1^Guangxi Botanical Garden of Medicinal Plants, Nanning 530023, Guangxi, China; ^2^Department of Pharmacy, Guigang City People's Hospital, The Eighth Affiliated Hospital of Guangxi Medical University, Guigang 537100, Guangxi, China; ^3^Guangxi Institute of Chinese Medicine and Pharmaceutical Science, Nanning 530022, Guangxi, China

## Abstract

Maoji Jiu (MJ), a medicinal wine, has been used commonly by the Chinese to enrich and nourish the blood. In this study, the aim is to examine the hematopoietic function of MJ and investigate its hematopoietic regulation mechanism. Thirty-six female Sprague-Dawley rats (200 ± 20 g) were randomly divided into six groups with six rats in each group. The blood deficiency model was induced by injecting hypodermically with N-acetylphenylhydrazine (APH) and injecting intraperitoneally with cyclophosphamide (CTX), and treatment drugs were given by oral gavage twice a day for continuous 10 days from the start of the experiments. The administration of MJ improved the levels of white blood cells (WBCs), red blood cells (RBCs), hemoglobin (HGB), and hematocrit (HCT) in the blood deficiency model rats. Hematopoietic effect involves regulating the antioxidant activity in the liver and the levels of Bcl-2, Bax, erythropoietin (EPO), transforming growth factor-beta-1 (TGF-*β*1), and macrophage colony-stimulating factor (M-CSF) mRNA in spleen tissues to enhance extramedullary hematopoiesis. This study suggests that MJ has a beneficial effect on blood deficiency model rats.

## 1. Introduction

Anemia, the most common disease of blood, is a condition associated with an insufficient number of healthy red blood cells resulting in lower hemoglobin levels. The disease of blood is often induced by increased erythrocyte damage, decreased or faulty red blood cell (RBC) production, and massive blood loss [1]. Insufficient blood supply to the liver and spleen is induced by blood deficiency directly, resulting in liver and spleen injuries that affect hemopoiesis in reverse [2]. The physiological conditions, such as menstruation, pregnancy, and postpartum, often occur in the female, and therefore, the anemic syndrome is more common in women than men and widely affects women's health. In addition, malignant tumor chemotherapy also has a common adverse reaction on hematopoietic function, which can exhibit leucopoenia and thrombocytopenia in patients [3]. Therefore, improving hematopoietic function in anemic patients and preventing the side effects of malignant tumor chemotherapy is a major goal of research in this field of medicine. It was reported that hemopoiesis is the process by which hematopoietic stem cells develop into mature blood cells through the stimulation of various cytokines in the hematopoietic microenvironment, including erythropoietin (EPO), transforming growth factor-beta-1 (TGF-*β*_1_), and macrophage colony-stimulating factor (M-CSF) [3]. Recently, it has been reported that many traditional Chinese medicines (TCMs) could treat blood deficiency because of their unique effect and reduced toxicity [4, 5].

Maoji Jiu (MJ) is a Chinese healthcare wine used commonly to treat women's ailments. It has notable effects on nourishing the blood and regulating menstruation, and these were recorded in the Chinese Pharmacopoeia (volume I, 1977 edition) [6]. It consists of *Centropus sinensis* (Hechiyajuan), *Angelica sinensis* Radix (Danggui), Chuangxiong Rhizoma (Chuanxiong), *Angelicae dahuricae* Radix (Baizhi), *Carthami flos* (Honghua), *Homalomenae* Rhizoma (Qiannianjian), *Poria cocos* (Fuling), *Paeoniae lactiflora* Pall. Radix (Chishao), Semen Persicae (Taoren), and some wine. The beneficial hematopoietic effect of the components of these TCMs has been proved in several previous studies [7–11]. Dating back to the late Qing Dynasty and the early Republic of China, MJ was first used in Guangdong and Guangxi provinces, and in the next few decades, it has been used extensively all over the world. Recently, MJ has a well-deserved reputation in China and Southeast Asian Nations because of its notable quality and efficacy.

Although MJ has been used commonly and widely for the treatment of blood deficiency for many years, there is no scientific study, to our best knowledge, on its potential pharmacological property. Thus, the study aims to explore the hematopoietic function of MJ and investigate its hematopoietic regulation mechanism, which will provide the basis for its clinical application. In this study, we copied blood deficiency model rats using N-acetyl phenylhydrazine (APH) and cyclophosphamide (CTX). Oxidative stress markers in liver tissue, apoptosis regulatory proteins, and hematopoietic growth factors (HGFs) in spleen tissue were measured to evaluate the protection and treatment of MJ in blood deficiency model rats. To evaluate the hematopoietic activity of MJ, Fufang Ejiao Jiang (FEJ), a drug that has been shown to possess powerful hematopoietic effects [12], was used as the positive control drug in this study.

## 2. Materials and Methods

### 2.1. Medical Materials and Extraction Protocol

Hechiyajuan was obtained from Pingnan, Guigang (Guangxi, China), and Danggui, Chuangxiong, Baizhi, Honghua, Qiannianjian, Fuling, Chishao, and Taoren were purchased from Yancheng Buyi Pharmacy Co. Ltd. (Jiangsu, China). In this study, the extraction protocol of MJ was used in a traditional and classical way which was recorded in the Chinese Pharmacopoeia (volume I, 1977 edition) [6]. Hechiyajuan was sacrificed, the viscera and plumage were wiped off, and then the whole body was dried in an oven. After steaming with a boiler for 15 minutes, Hechiyajuan (160 g) was placed in a glass jar, appropriate wine (the ethanol content was 40 percent) was poured into the jar (inundated with *Centropus sinensis*), and then the jar was sealed for 25 days. The rest of the medical materials, Danggui (160 g), Chuangxiong (160 g), Baizhi (160 g), Honghua (160 g), Qiannianjian (160 g), Fuling (20 g), Chishao (15 g), Taoren (15 g), and the remaining wine, with the total volume up to16800 ml, were added to the same jar. The jar was sealed again for additional 55 days and then filtered through gauze. The accumulated filtrate was evaporated with a rotary evaporator under a vacuum at 50°C. The ethanol content of the concentrate was adjusted to 40 percent (v/v), and the total volume was 8400 ml. Then, the MJ sample was obtained and stored at room temperature till further use.

### 2.2. Experimental Animals

Thirty-six female Sprague-Dawley rats (200 ± 20 g) were provided by Guangxi Medical University Laboratory Animal Centre (Nanning, China, License No. SCXK [Gui] 2014-0002). All rats were kept in a case at a temperature of 20–25°C and relative humidity of 40–60% under a 12 h light/dark cycle with free access to tap water and a standard diet. Rats were allowed to acclimatize for five days before the experiments. The animal experiments described in this study were approved by the Ethics Committee of Guangxi Botanical Garden of Medicinal Plants (no. 20170301).

### 2.3. Blood Deficiency Model Construction and Administration

After acclimatization, the rats were randomly divided into six groups, each with six rats. The groups are the control group (CG), model group (MG), FEJ group, high dose group of MJ (MJ-H), middle dose group of MJ (MJ-M), and low dose group of MJ (MJ-L). Blood deficiency model construction referred to Zeng's method [13]. The rats in the model group and FEJ, MJ-H, MJ-M, and MJ-L groups were hypodermically injected with 2% APH (Shanghai Ziyi Reagent Factory, Shanghai, China) saline solution on the 1st and 4th day at the dose of 20 mg/kg and 10 mg/kg, respectively; 2 hours after injections on the 4th day, the rats were intraperitoneally injected with CTX (Baxter Oncology GmbH, Halle, Germany) saline solution at a dose of 15 mg/kg for continuous four days. The rats in the control group were hypodermically or intraperitoneally injected with an equal volume of normal saline (NS). Thus, the blood deficiency model was created. The rats in MJ-H, MJ-M, and MJ-L groups were intragastrically given 10 ml/kg, 5 ml/kg, and 2.5 ml/kg body mass of MJ, respectively, twice a day for 10 continuous days. The rats in the FEJ group were intragastrically given 6 ml/kg body mass of FEJ twice a day for 10 continuous days. Simultaneously, the rats in the control group and model group were given an equal volume of NS by oral gavage. All rats were sacrificed on the 11th day. Blood, thymus, spleen, liver, and lung samples were obtained for further examination.

### 2.4. Blood Routine Examination and Visceral Index Calculation

The blood samples of inner canthus 0.5 ml were collected to assay the routine blood tests using a SysmexXT-2000iv full-automatic blood cell analyzer (Sysmex Company, Japan), which included white blood cell (WBC), RBC, hemoglobin (HGB), hematocrit (HCT), and platelets (PLT) levels. The visceral index, including the thymus, spleen, liver, and lung, was calculated according to the formula: visceral index = visceral weight (mg)/rat weight (g).

### 2.5. Assessment of Antioxidant Activity and Lipid Peroxidation

The markers of oxidative stress, including SOD, T-AOC, and MDA, were detected using commercially available kits (Nanjing Jiancheng Bioengineering Research Institute, Nanjing, China) according to the manufacturer's protocol. In brief, liver tissues were homogenized on ice with NS (10% homogenates). Then, the homogenates were centrifuged at 2500 × g for 10 min at 4°C to obtain the supernatants, which were used for detecting the activities of SOD and T-AOC and the content of MDA by employing a commercially available kit according to the manufacturer's protocol.

### 2.6. Histopathological Examination

The spleen tissue was obtained, and a piece was immediately fixed in phosphate-buffered saline containing 4% formaldehyde. After fixation, the sample was embedded in paraffin, sectioned in 5 *μ*m thick sections, and mounted on slides. The paraffin-embedded sections were rehydrated using xylene and alcohol series and then stained with hematoxylin and eosin (H&E) to observe histological changes, and these were evaluated using optical microscopy (Olympus).

### 2.7. Immunohistochemistry

The paraffin-embedded sections of spleen samples were also rehydrated using xylene and alcohol series and then labeled separately with rat monoclonal antibody Bcl-2 (1 : 100, Bioss, Beijing, China) and Bax (1 : 1000, Santa Cruz, USA) as the primary antibodies and streptavidin peroxidase as a secondary antibody, respectively, to observe the presence of these antigens under optical microscopy (Olympus, China), and Image-Pro Plus 6.0 was used to analyze the Bcl-2 and Bax expressions semiquantitative.

### 2.8. Cytokine Secretion

The spleen tissues together with 10 times NS were homogenized using a homogenizer, and then the total proteins of the homogenates were detected using a nucleic acid detector (Thermo, America). The EPO concentrations in the homogenates were assayed using the Enzyme-Linked Immunosorbent Assay (ELISA) kits (Wuhan Boster Bio-Engineering Co. Ltd, Wuhan, China) according to the manufacturer's instructions.

### 2.9. Real-Time PCR Analysis

Total RNA from spleen tissues was extracted using a Trizol reagent kit (Takara, Japan) according to the kit instructions. The RNA concentration was determined by measuring the optical density at 260 nm. Complementary DNA (cDNA) was synthesized with a PrimeScript™ II reverse transcriptase reagent kit (Takara, Japan) from 1 *μ*g of total RNA. Quantitative Real-Time PCR was performed on a 7300 Real-Time PCR detection system (Applied Biosystems, Foster City, CA, USA) using a SYBR® Green PCR kit (Takara, Japan). The PCR procedure was set as 95°C, 30 s, 95°C, 5 s, and 60°C, 31 s, 40 cycles. Sample cDNAs (equivalent to 2 *μ*g of total RNA) were regarded as templates with gene-specific primers. The PCR primer sequences were set as follows: TGF-*β*_1_: 5′-CATTGCTGTCCCGTGCAGA-3′(forward) and 5′-AGGTAA -CGCCAGGAATTGTTGCTA-3′ (reverse); M-CSF: 5′-GAATACTGAACCTGCCTGCTGAA-3′(forward) and 5′-AGGCCAGCTCAGTGCAAGAA-3′ (reverse); *β*-actin: 5′-GGAGATTACTGCCCTGGCTCCTA-3′ (forward) and 5′-GACTCATCGTACTCCTGCTTGCTG-3′ (reverse). *β*-Actin was used as the housekeeping gene. The expressions of target mRNAs were measured using the 2^−ΔΔCt^ method and normalized to *β*-actin in arbitrary units.

### 2.10. Statistical Analysis

Statistical analysis was performed using SPSS 16.0, and the results are expressed as mean ± SD, where SD represents standard deviation. Statistical differences between groups were determined using a one-way analysis of variance. A value of *P* < 0.05 was considered to be statistically significant.

## 3. Results

### 3.1. Appearance and Histopathological Examination of the Blood Deficiency Model Rats

**Figure 1:**
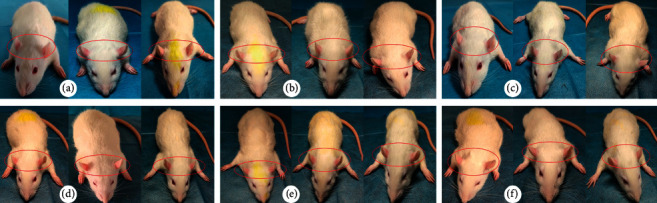
Effect of MJ on the appearance of the blood deficiency model rats. (a) Control group, (b) model group, (c) FEJ group, (d) MJ-H group, (e) MJ-M group, and (f) MJ-L group.

The appearances of rats were changed after being given APH and CTX. As shown in Figure 1, the rats in the control group appeared to be in good condition; their ears, noses, faces, and tails were pink, and their hairs were tight and glossy. However, the rats in the model group appeared to be in bad condition; their hairs were fluffy and shed easily, and their ears, noses, faces, and feet were pale. Particularly, the ears of the model group were easily observed. After administration, the above features of the rats in the FEJ, MJ-H, MJ-M, and MJ-L groups were similar to those in the control group. Among those groups, the MJ-H was the closest.

**Figure 2:**
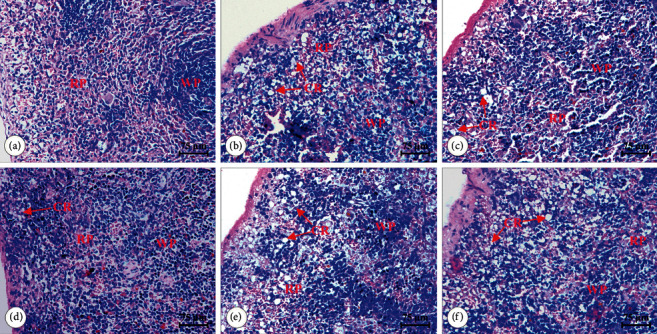
Effects of MJ on the histological structure in spleen tissues of blood deficiency model rats. (a) Control group, (b) model group, (c) FEJ group, (d) MJ-H group, (e) MJ-M group, and (f) MJ-L group (H&E × 200, scale bar = 75 *μ*m).

Histopathological changes in spleen sections were observed and evaluated by H&E staining (Figure 2). The spleen tissues in the rats of the control group showed a normal spleen architecture clearly with regular distribution of red pulps (RP) and white pulps (WP), tight arrangement of lymphocytes, and no obvious existence of segmentation and differentiation. In contrast, the structure of white pulps in splenocytes of the model group was destroyed. Crypta (CR) increased, and lymphocytes decreased. In the FEJ treatment group, the rats appeared to be better, with less connective tissue and more lymphocytes. These characteristics of rats in the model group were also observed after treatment with MJ, but they were significantly improved compared with those in the model group.

### 3.2. Effects of MJ on Peripheral Routine Blood and Visceral Index of the Blood Deficiency Model Rats

The indexes of peripheral routine blood directly reflect the curative effect of replenishing blood. As shown in Table 1, the WBC, RBC, HGB, HCT, and PLT levels in the model group decreased significantly compared with those in the rats in the control group (*P* < 0.01), suggesting that the model was copied successfully. The WBC, RBC, HGB, and HCT levels in MJ and FEJ treatment groups showed an increasing trend compared with those in the rats in the model group.

The changes in visceral indexes can reflect the injury of organs in the blood deficiency model rats. As shown in Table 2, the liver index, thymus index, and spleen index in rats in the model group were reduced significantly compared with those in the control group (*P* < 0.01). Compared with the model group, the liver index and spleen index in the MJ and FEJ treatment groups showed a decreasing trend, and the thymus index in the MJ and FEJ treatment groups showed an obvious increasing trend. The lung index in the control and treatment groups had no statistical difference compared with the rats in the model group.

### 3.3. Effects of MJ on Antioxidant Activity and Lipid Peroxidation of the Blood Deficiency Model Rats

The markers of antioxidant capacity and lipid peroxidation mainly include SOD, T-AOC, and MDA. As shown in Table 3, the activity of T-AOC presented a significant increase in rats in the control group and the blood deficiency model rats treated with MJ or FEJ compared with the rats in the model group (*P* < 0.05). In addition, the level of MDA in the rats in the model group decreased significantly compared with those in the control group (*P* < 0.01). Compared with the model group, the levels of MDA in the rats in the MJ-H, MJ-M, and FEJ groups reduced significantly (*P* < 0.05), but there was little impact on this marker in the MJ-L group. The activity of SOD in the rats in the model group showed a small decrease compared with the other groups, but this was not statistically significant.

### 3.4. Effects of MJ on Apoptosis Regulatory Proteins and HGFs in Spleen Tissues of Blood Deficiency Model Rats

**Figure 3:**
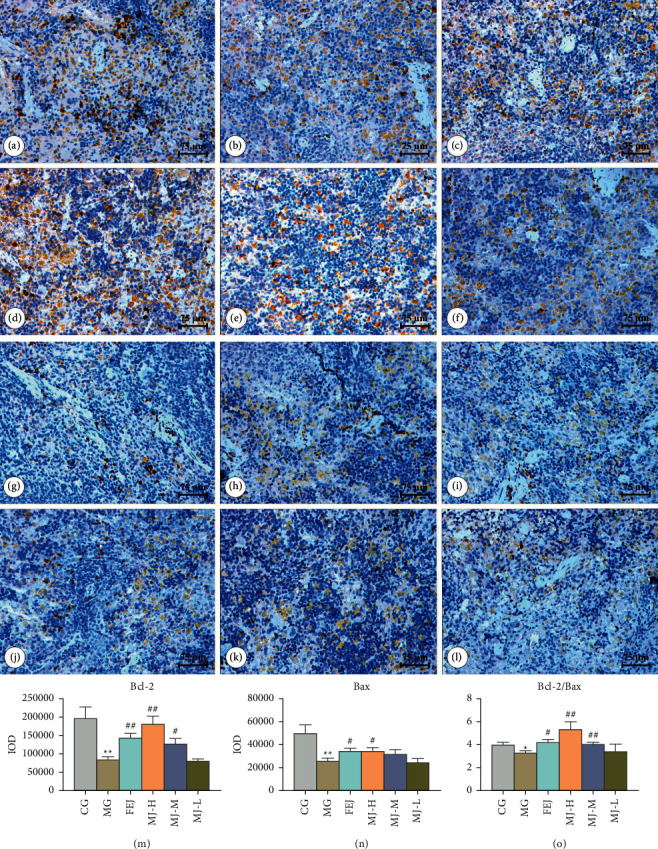
Effects of MJ on the expressions of Bcl-2 and Bax in spleen tissues of blood deficiency model rats. Representative photomicrographs of the histological slides of Bcl-2 in rat spleens (a∼f). Representative photomicrographs of the histological slides of Bax in rat spleens (g∼l). The means ± SD of the average integral optical density (IOD) of the splenic Bcl-2 and Bax proteins from the experimental rats (m∼o). (a and g) Control group, (b and h) model group, (c and i) FEJ group, (d and j) MJ-H group, (e and k) MJ-M group, and (f and l) MJ-L group (immunohistochemistry × 200, scale bar = 75 *μ*m). ^*∗*^*P* < 0.05 and ^*∗∗*^*P* < 0.01 compared with the control group; ^#^*P* < 0.05 and ^##^*P* < 0.01 compared with the model group.

Firstly, we found that the expressions of Bcl-2 and Bax and the Bcl-2/Bax ratio decreased significantly (*P* < 0.05) in blood deficiency model rats that were induced by APH and CTX. After the administration of MJ and FEJ, Bcl-2 expressions and the Bcl-2/Bax ratio of the rats in the FEJ, MJ-H, and MJ-M groups increased significantly (*P* < 0.05), and Bax expressions of FEJ and MJ-H groups increased significantly (*P* < 0.05) compared with those in the model group (Figure 3).

**Figure 4:**
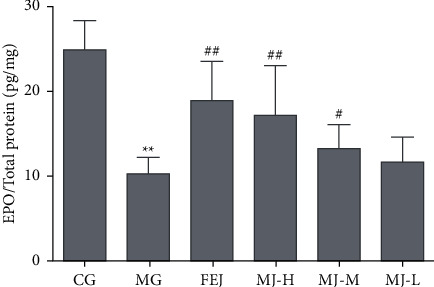
Effects of MJ on EPO level of spleen tissues of blood deficiency model rats. ^*∗*^*P* < 0.05 and ^*∗∗*^*P* < 0.01 compared with the control group; ^#^*P* < 0.05 and ^##^*P* < 0.01 compared with the model group.

Secondly, we detected the EPO levels in spleen tissues of blood deficiency model rats, and the results are shown in Figure 4. The EPO level in the model group decreased significantly (*P* < 0.01) compared with that in the control group. However, the EPO levels of the FEJ and MJ-H groups increased significantly (*P* < 0.05), and the EPO levels of MJ-M and MJ-L groups also showed an increasing trend compared with those in the model group.

**Figure 5:**
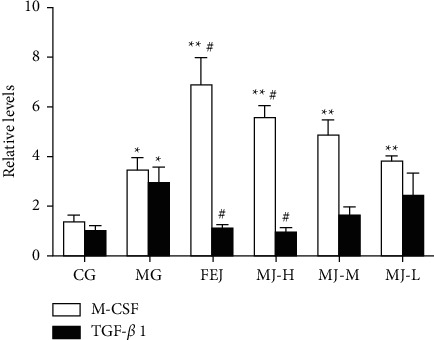
Effects of MJ on M-CSF and TGF-*β*1 mRNA level of spleen tissues of blood deficiency model rats. ^*∗*^*P* < 0.05 and ^*∗∗*^*P* < 0.01 compared with the control group; ^#^*P* < 0.05 and ^##^*P* < 0.01 compared with the model group.

Finally, we determined and compared the M-CSF and TGF-*β*_1_ mRNA levels in spleen tissues of blood deficiency model rats (Figure 5). Compared with the control group, the M-CSF mRNA levels in the other groups decreased significantly (*P* < 0.05), but the TGF-*β*_1_ mRNA level in the model group increased significantly (*P* < 0.05). Compared with rats in the model group, the M-CSF mRNA levels in FEJ and MJ-H groups increased significantly (*P* < 0.05), but the TGF-*β*_1_ mRNA levels in FEJ and MJ-H groups decreased significantly (*P* < 0.05). These results demonstrated that MJ could regulate hematopoiesis via M-CSF and TFG-*β*_1_, with a similar effect as FEJ if the MJ dose is high.

## 4. Discussion

In modern medicine, the decreased level of hemoglobin is commonly considered a blood deficiency, containing aplastic anemia, iron deficiency anemia, and blood loss anemia [14]. It was shown that the blood deficiency model induced by APH and CTX was more similar to the internal environment in blood deficiency [9]. APH, as a strong oxidizing agent, decreases the production of glutathione and destroys the stability of red blood cell membranes [15]. CTX, as an antitumor drug commonly used in the clinic, has a primary adverse effect on immune organs and the total number of reduced RBC, WBC, and PLT in peripheral blood [16]. Thus, we induced rats into a blood deficiency model by APH and CTX and then detected peripheral blood routine parameters. The results showed that the WBC, RBC, HGB, and HCT levels in the MJ treatment groups were higher than those in the model group (Table 1), which indicated that MJ could improve the peripheral blood routine of the blood deficiency rats induced by APH and CTX.

Having confirmed that MJ could improve hematopoietic function, we investigated its mechanism further. Blood deficiency syndrome may be connected with the weak antioxidant capacity of the body [17–19]. The activities of T-AOC and the level of MDA in the liver are commonly detected to assess the antioxidative property in previous studies [13, 20, 21]. In our study, the decreased activity of T-AOC and the increased level of MDA in liver tissues in the model group were observed (Table 3), which was consistent with the blood deficiency model mice in previous studies [10, 21, 22]. The variational tendency of the two markers was reversed by MJ treatment, indicating that MJ could improve the antioxidative property via T-AOC and MDA in blood deficiency model rats.

Bcl-2 and Bax genes are two apoptosis regulatory proteins that belong to the Bcl-2 gene family, which plays an important role in regulating cell apoptosis. Previous studies showed that cell viability can be monitored by measuring the ratio of Bcl-2 and Bax after the activation of apoptosis [23–25]. In this study, splenomegaly, injury, and apoptosis of spleen cells in the model group were observed, which might be associated with the insufficient blood supply to the spleen when the body was in a state of blood deficiency [2]. The splenomegaly, injury, and apoptosis of spleen cells could inhibit the hematopoietic function of the body in a certain degree [2]. We also observed that when MJ was administered, this phenomenon in the treatment groups was alleviated. To better elucidate the cause behind the injury and apoptosis, we used immunohistochemical methods to determine the expressions of Bcl-2 and Bax in the spleen. The Bcl-2/Bax ratio in the model group decreased significantly (Figure 3), and the changes in the two markers were confirmed to be associated with splenic injury [26, 27]. The Bcl-2/Bax ratio increased after the treatment with MJ, suggesting that MJ may reduce spleen cell injury and apoptosis by regulating the expressions of Bcl-2 of and Bax.

Hematopoietic growth factors from spleen cells, bone marrow stromal cells, and other cells [28] play an important role in the growth and differentiation of various blood cells [3]. EPO and M-SCF are two essential HGFs that participate in hematopoietic regulation [7, 29]. EPO regulates the erythropoiesis production and stimulates the proliferation of early erythroid precursors and the differentiation of late erythroid precursors [30]. TGF-*β*_1_, a member of TGF-*β*, plays an important role in regulating hematopoiesis. Hematopoiesis could be inhibited when it is at a high level [31]. In our study, the increased expressions of M-CSF mRNA in the blood deficiency model rats were induced by APH and CTX (Figure 5), which may be related to a self-healing mechanism when rats were in a blood deficiency status. However, the expressions of M-CSF mRNA in the MJ treatment groups were higher than those in the model group. The content of EPO in the model group decreased relative to the control group (Figure 4), which was consistent with the decreased content of EPO in blood deficiency model mice [3]. However, the level of TGF-*β*_1_ mRNA in the model group increased compared with that in the control group, which was in accordance with the increased level of TGF-*β*_1_ mRNA in the mouse model of myelofibrosis in the spleen [31]. The changes in EPO and TGF-*β*_1_ mRNA were reversed after the treatment of MJ. These results indicate that MJ may increase hematopoietic activity by increasing the EPO and M-CSF mRNA levels and decreasing the TGF-*β*_1_ mRNA.

Anemia is a global problem. Statistics from a report shows that the number of anemia cases worldwide had exceeded 1.8 billion, accounting for about 23% of the world's total population [32]. MJ has the dual properties of food and medicine and notable effects on nourishing the blood, which may be more likely chosen by people suffering from anemia. In our study, the hematopoietic function of MJ was preliminarily evaluated, and the results confirmed that MJ has a notable hematopoietic efficacy. However, studies on MJ are still in a backward stage, which restricts its further popularization and application in hematopoietic function. Therefore, more attention should be paid to studies on the blood-enriching effect of MJ in order to provide a safe and effective agent for patients with blood deficiency. The findings of our study have to be seen in light of some limitations. Firstly, bone marrow, one of the hematopoietic organs, is considered one of the gold standard markers for evaluating hematopoietic injury or deficiency in animals in previous studies [33–35]. However, the markers of bone marrow in the blood deficiency model rats were not detected in our study. Secondly, the active constituents of the hematopoietic function in MJ playing a crucial role in the further drug development were not filtrated and identified. Moreover, the safety of Maoji Jiu in animals or humans remains unclear. These limitations are planned to be the potential scopes of MJ in our future studies.

## 5. Conclusions

In our study, MJ shows certain hematopoietic function *in vivo* in blood deficiency model rats. The findings indicated that MJ could effectively protect rats against blood deficiency, which was probably related to improving antioxidant activity in liver, alleviating spleen injury, and regulating HGFs in the spleen. Thus, we propose that MJ could be used as a potential TCM for treating blood deficiency.

## Figures and Tables

**Table 1:** The changes of peripheral blood routine in blood deficiency model rats (mean ± SD, *n* = 6).

Group	WBC/×10^9^/L	RBC/×10^12^/L	HGB/g·L^−1^	HCT/%	PLT/×10^9^/L
CG	9.15 ± 1.00	7.18 ± 0.31	135.67 ± 6.12	42.67 ± 1.71	929.0 ± 140.04
MG	2.89 ± 0.45^*∗*^	3.47 ± 0.32^*∗*^	89.17 ± 10.82^*∗*^	31.17 ± 2.72^*∗*^	646.0 ± 62.66^*∗*^
FEJ	3.63 ± 0.48^#^	3.99 ± 0.15^##^	101.33 ± 5.35^#^	33.57 ± 2.96	810.5 ± 159.79^#^
MJ-H	3.74 ± 0.47^##^	4.20 ± 0.37^##^	105.83 ± 6.82^##^	36.57 ± 3.14^##^	656.0 ± 168.00
MJ-M	3.55 ± 0.54^#^	3.95 ± 0.34^#^	100.17 ± 4.22^#^	35.33 ± 2.18^#^	679.8 ± 125.57
MJ-L	3.16 ± 0.88	3.80 ± 0.16^#^	94.83 ± 6.15	33.33 ± 1.53	639.7 ± 99.26

Note: ^*∗*^*P* < 0.01 compared with the control group; ^#^*P* < 0.05 and ^##^*P* < 0.01 compared with the model group.

**Table 2:** The visceral index changes in blood deficiency model rats (mean ± SD, *n* = 6).

Group	Liver index (mg/g)	Thymus index (mg/g)	Spleen index (mg/g)	Lung index (mg/g)
CG	25.97 ± 1.30	1.79 ± 0.21	2.35 ± 0.12	5.20 ± 0.55
MG	32.52 ± 3.01^*∗*^	0.74 ± 0.05^*∗*^	5.41 ± 0.47^*∗*^	5.48 ± 0.35
FEJ	29.23 ± 1.65^#^	0.87 ± 0.12^#^	5.13 ± 1.05	5.16 ± 0.44
MJ-H	29.75 ± 0.52^#^	0.94 ± 0.12^##^	4.84 ± 0.40^#^	5.35 ± 0.23
MJ-M	30.59 ± 0.98	0.87 ± 0.09^#^	4.91 ± 0.25^#^	5.33 ± 0.39
MJ-L	30.51 ± 1.62	0.81 ± 0.18	5.24 ± 1.04	5.32 ± 0.69

Note: ^*∗*^*P* < 0.01 compared with the control group; ^#^*P* < 0.05 and ^##^*P* < 0.01 compared with the model group.

**Table 3:** Effects of MJ on the activity of SOD and T-AOC and the level of MDA in livers of blood deficiency model rats (mean ± SD, *n* = 6).

Group	SOD (U/mg prot)	T-AOC (U/mg prot)	MDA (nmol/mg prot)
CG	197.50 ± 7.92	12.51 ± 1.35	9.45 ± 1.05
MG	195.69 ± 5.78	8.68 ± 1.50^*∗∗*^	15.44 ± 2.11^*∗∗*^
FEJ	200.53 ± 12.27	10.54 ± 0.85^#^	12.98 ± 1.02^#^
MJ-H	196.04 ± 11.24	12.47 ± 1.37^##^	12.40 ± 1.08^##^
MJ-M	195.43 ± 6.47	11.55 ± 0.76^##^	13.25 ± 0.90^#^
MJ-L	194.50 ± 15.42	11.36 ± 1.29^##^	13.62 ± 1.22

Note: ^*∗*^*P* < 0.01 compared with the control group; ^#^*P* < 0.05 and ^##^*P* < 0.01 compared with the model group.

## Data Availability

The data used to support the findings of this study are available from the corresponding author upon request.

## Abbreviations

MJ: Maoji Jiu
APH: N-Acetylphenylhydrazine
CTX: Cyclophosphamide
HGFs: Hematopoietic growth factors
WBC: White blood cell
RBC: Red blood cell
HGB: Hemoglobin
HCT: Hematocrit
PLT: Platelet
EPO: Erythropoietin
TGF-*β*1: Transforming growth factor-beta-1
M-CSF: Macrophage colony-stimulating factor
TCMs: Traditional Chinese medicines
FEJ: Fufang Ejiao Jiang
SOD: Superoxide dismutase
T-AOC: Total antioxidant activity
MDA: Malondialdehyde
ELISA: Enzyme-Linked Immunosorbent Assay
CG: Control group
MG: Model group
MJ-H: High dose group of Maoji Jiu
MJ-M: Middle dose group of Maoji Jiu
MJ-L: Low dose group of Maoji Jiu
NS: Normal saline
H&E: Hematoxylin-eosin
PCR: Reverse transcription-polymerase chain reaction
cDNA: Complementary DNA
RP: Red pulps
WP: White pulps
CR: Crypta.
